# Cultivar and Tree Density As Key Factors in the Long-Term Performance of Super High-Density Olive Orchards

**DOI:** 10.3389/fpls.2016.01226

**Published:** 2016-08-23

**Authors:** Concepción M. Díez, Juan Moral, Diego Cabello, Pablo Morello, Luis Rallo, Diego Barranco

**Affiliations:** ^1^Departamento de Agronomía, Campus de Excelencia Internacional Agroalimentario (ceiA3), Universidad de CórdobaCórdoba, Spain; ^2^Department of Plant Pathology, Kearney Agricultural Research and Extension Center, University of California, DavisDavis, CA, USA

**Keywords:** *Olea europaea* L., orchard design, oil yield, biennial bearing, hedgerows

## Abstract

Super high-density (SHD) olive orchards are rapidly expanding since the first plantation was set up in Spain in the 1990s. Because there are no long-term studies characterizing these systems, it is unknown if densities above a certain threshold could trigger competition among fully-grown trees, compromising their development. Over 14 years we have evaluated the performance of the major olive cultivars currently planted in SHD systems (“Arbequina,” Arbequina IRTA-i·18, “Arbosana,” “Fs-17,” and “Koroneiki”) and nine SHD designs ranging from 780 to 2254 trees ha^−1^ for the cultivar “Arbequina.” Remarkably, the accumulated fruit and oil production of the five cultivars increased linearly over time. Our data indicated the favorable long-term performance of the evaluated cultivars with an average annual oil production of 2.3 t ha^−1^. Only “Fs-17” did not perform well to the SHD system in our conditions and it yielded about half (1.2 t ha^−1^) of the other cultivars. In the density trial for “Arbequina,” both fruit and oil accumulated production increased over time as a function of tree density. Thus, the accumulated oil yield ranged from 16.1 t ha^−1^ for the lowest density (780 trees ha^−1^) to 29.9 t ha^−1^ for the highest (2254 trees ha^−1^). In addition, we note that the accumulated production per surface unit showed a better correlation with the hedgerow length than the tree density. Thus, the current planting designs of SHD olive orchards can be further improved taking this parameter into account. Despite observations that some irregular patterns of crop distribution have arisen, our olive hedgerows are still fully productive after 14 years of planting. This result contradicts previous experiences that showed declines in production 7 or 8 years after planting due to high vigor, shading, and limited ventilation.

## Introduction

Traditional Mediterranean olive orchards are designed in the form of extensive plantations with densities of 70–80 trees ha^−1^. However, hedgerow orchards are gaining popularity worldwide; these are also known as super high-density (SHD) systems, narrow hedgerows with an average of 1500–2000 trees ha^−1^. Compared to traditional systems, these SHD orchards have several advantages, including rapid mechanized harvesting and pruning, high crop levels, and early bearing. These features make SHD systems profitable, and thus they are commonly chosen for new plantations, especially in non-traditional olive-growing countries, such as Argentina, Australia, and the USA (Rallo et al., 2013; Connor et al., 2014). Currently, there are more than 100,000 ha of SHD olive orchards worldwide, and this number is growing (Rius and Lacarte, 2015).

A major drawback associated with SHD systems is that they require high initial investments; in addition, limited information has led to uncertainties concerning their optimum design, suitable cultivars, management strategies, and longevity (Freixa et al., 2011; Rallo et al., 2013; Connor et al., 2014). This lack of knowledge stems from the relatively recent development of SHD systems for olives and the sparseness of scientific and technical research. SHD systems were first applied to other fruit crops including apples, peaches, and cherries (Ryugo and Mikuckis, 1969; Heinicke, 1975; DeJong and Doyle, 1985). In these crops, the spread of SHD systems was enhanced by the development of new training systems, low-vigor cultivars, and dwarfing rootstocks (Webster, 1993; Day et al., 2005; Robinson, 2005; Robinson et al., 2013; Musacchi et al., 2015).

The first SHD olive orchards were established during the 1990s in Spain and were primarily promoted by private companies (Rius and Lacarte, 2015). Since then, commercial olive production has advanced more rapidly than scientific research regarding the response of olives to SHD. Lately, however, technical research has made significant strides in characterizing and evaluating the factors that affect the performance of SHD olive orchards.

The success of olive SHD systems in Mediterranean areas largely depends on careful management of water and nutrients to control the vigor of the trees, as well as the optimum combination of a suitable cultivar and appropriate orchard design (Rallo et al., 2013; Connor et al., 2014). The two latter factors are especially critical in rain fed SHD olive orchards, which are currently being planted in areas with low to medium rainfall such as the Guadalquivir Valley in southern Spain. The use of low vigor cultivars is also crucial in SHD systems, because shadowing problems can arise more quickly for vigorous cultivars, compromising the long-term health of the orchard (Rallo et al., 2013).

Only a few traditional olive cultivars meet the low vigor required by SHD systems. In addition, no rootstocks, able to modify the scion growth habit, have been developed for olive because, in contrast to other fruit crops, olive cultivars primarily grow from their own roots (Barranco, 2010). The traditional cv. “Arbequina” is by far the dominant cultivar used in SHD orchards given its low vigor, high and stable productivity, and fruity oil (Barranco et al., 2000; Rius and Lacarte, 2015). SHD systems also include two other traditional cultivars that have low or moderate vigor, “Arbosana” and “Koroneiki” (Barranco et al., 2000; Tous et al., 2011; Rallo et al., 2013). The cultivars “Arbequina” and “Arbosana” share a compact architectural pattern that increases yield efficiency in SHD conditions compared to other cultivars (Rosati et al., 2013). “Koroneiki” is also a highly productive cultivar that yields high-quality oils, but it is more vigorous than “Arbequina” and “Arbosana” (Barranco et al., 2000). However, the extent to which the different vigor and growth habits of the “Arbequina,” “Arbosana,” and “Koroneiki” cultivars will affect the long-term performance of SHD orchards is unknown.

The distance between trees and rows primarily defines the orchard design, which is also constrained by the availability of irrigation water and the orientation of the rows (Pastor et al., 2007; Rallo et al., 2013; Rosecrance et al., 2015; Trentacoste et al., 2015a,b). An optimum orchard design should favor maximum yields without compromising the management of the orchard, for example, by still allowing access by harvester machines and other vehicles. High yields are significantly influenced by the illumination of the hedgerows, which is primarily determined by the ratio (D/A) of canopy depth to free alley width (i.e., the row spacing minus the hedgerow width). As with other fruit crops, in N-S oriented rectangular olive orchards, oil yield is maximized when the canopy depth equals the free alley width (D/A = 1; Smart et al., 1990; Robinson, 2011; Connor and Gómez-del-Campo, 2013). During the first years after planting, the oil yield increases linearly with tree density (De la Rosa et al., 2007; León et al., 2007; Larbi et al., 2011, 2012; Farinelli and Tombesi, 2015). However, it is still unknown if densities above a certain threshold can trigger competition among fully-grown trees, compromising their development and production and ultimately the lifespan of the olive orchard. Long-term comparative trials are necessary to resolve this debate (Rallo et al., 2013).

To date, all the studies have characterized SHD olive systems during the first 4–7 years after planting (Hermoso et al., 2004; Camposeo and Godini, 2010; Larbi et al., 2011, 2012; Tous et al., 2011; Proietti et al., 2014; Farinelli and Tombesi, 2015; Trentacoste et al., 2015a); and no long-term evaluations that exceed this period have been published. This situation is especially striking given the global spread of SHD olive orchards (Rius and Lacarte, 2015). Overall, this study aims to address this gap in knowledge by evaluating SHD orchard designs and cultivars in two comparative field trials over a 14 year period. It also tries to address the following specific questions. First, which are the best-suited cultivars for SHD olive orchards? Second, what is the average oil production for this kind of orchard design over a long-term period? Does the production decline over time? Finally, what is the effect of increasing tree density on production? Does the tree density affect the characteristics of the cultivars?

Preliminary results of these two trials were published 7 years after planting, providing the first description of the early performance of SHD olive orchards (De la Rosa et al., 2007; León et al., 2007). Now, after 14 years, the two trials are still fully productive. Despite some irregular patterns of crop distribution were detected, the trials did not present the problems previously reported for SHD orchards at 7 years after planting (Pastor et al., 2007). The selection of the cultivar, the orchard design as well as, the control of the tree vigor, emerge as critical factors to guarantee the production and longevity of SHD olive orchards.

## Materials and methods

### Trial location and management

Two trials were conducted over 14 years to assess the response of five olive cultivars, “Arbequina,” Arbequina IRTA-i·18, “Arbosana,” “Fs-17,” and “Koroneiki,” to SHD conditions and determine the optimum SHD design for the cultivar “Arbequina.” Both trials were located in Pedro Abad, Cordoba, in southern Spain (37°57′32″N, 4°27′44″W and 162 m a.s.l.) in a plot owned by the company Todolivo S.L. The climate of the area is Mediterranean; the average annual precipitation from 2001 to 2013 was 566 mm, and there was marked summer drought with less than 30 mm of precipitation from June to September. The average potential evapotranspiration (ETP) from 2001 to 2013 was 938 mm, and the average annual, maximum and minimum temperatures were 17.5, 24.9, and 10.3°C, respectively (Tragsa, 2015). The area of study was an alluvial plain formed by deposition of Guadalquivir river and the soil was classified as a Typic Xerofluvent (Soil Survey Staff, 2015). The soil was ~1.5 m deep and with a sandy-loam Ap upper horizon (0–35 cm), pH = 8.1, and 1.2% of organic matter content. The available phosphorous (P) and potassium (K) contents in the soil were 19.2 and 418 mg kg^−1^, respectively. Field capacity and permanent wilting point were 0.23 and 0.07 m^3^ m^−3^, respectively (Testi et al., 2004). These latter values were not determined for the experimental plots but for a geographically nearby olive orchard with similar soil characteristics.

The trials were set up in March of 2000, when 50 cm-tall, self-rooted olive trees were planted. The rows were oriented north to south, and the trees were trained to a central leader to promote hedgerow formation, after which they were pruned annually with the exception of 2011 and 2013. The olive trees were also pruned to remove shoots up to 50 cm off the ground and lateral branches extending into alleys. Automatic lateral and top pruning was used to fit the trees to the harvester machine. The sites were irrigated from May to September applying 100 m^3^ per ha per week (2000 m^3^ of water per year) using drip irrigation; during some years the same irrigation practice was extended until the end of October. Foliar fertilization was applied four times per year during the months of November (after harvesting), March, May and September, with 2% potassium nitrate. No fertilizers were intentionally applied to the soil but the nitrate contamination of the irrigation water reached values as high as 87 mg/l, with a median in the area close to 79 mg/l (López-Gutierrez et al., 2012). Therefore, irrigation approximately supplied 174 kg of N ha^−1^ year^−1^. Pest control and soil management practices were applied according to the conventional practices recommended for SHD olive orchards (Rius and Lacarte, 2015). Three extreme climatic episodes affected the trials during the 14 years of evaluation. First, unusually heavy and prolonged rainy periods caused waterlogging during the winters of 2003–2004 and 2010–2011, when 345 and 522 mm of rain fell from October to December, respectively. Second, severe frosts damaged the trees during the winters of 2004–2005 and 2011–2012, with temperatures below −9° and −6°C, respectively. Finally, high temperatures (above 36°C for 10 days) in May of 2012 caused ovary abortion and yield loss.

### Experimental design

The first trial was aimed to evaluate the performance of the cvs. “Arbequina,” Arbequina IRTA-i·18, “Arbosana,” “Fs-17,” and “Koroneiki” in SHD systems. “Arbequina” and “Arbosana” are traditional cultivars from Catalonia, Spain, and “Koroneiki” is a major traditional cultivar from Greece (Barranco et al., 2000). Arbequina IRTA-i·18 is a clone of “Arbequina” selected in Catalonia (Tous et al., 1998), and “Fs-17” is derived from a cross of “Frantoio” in open pollination (Fontanazza et al., 1998). The olive trees were planted with a distance of 3.75 m between rows and 1.35 m between the trees (1975 trees ha^−1^). The experimental design was a randomized complete block with four replicates. Every block was composed of four rows with 40 trees of each cultivar as the experimental unit.

The second trial focused on testing the performance of “Arbequina” in nine designs with different tree densities, including 780, 909, 952, 1143, 1203, 1481, 1569, 2000, and 2254 olive trees per ha. These densities were, respectively, the result of the following combinations of distances between rows and trees within rows (m): 5.70 × 2.25, 5.50 × 2.00, 5.25 × 2.00, 5.00 × 1.75, 4.75 × 1.75, 4.50 × 1.50, 4.25 × 1.50, 4.00 × 1.25, and 3.55 × 1.25. The experimental design of the trial was a randomized complete block with four replicates, where each experimental unit consisted of a 40 m-long row with 18–32 trees (depending on the density).

### Experimental variables

Both trials were monitored from 2002 to 2013 (henceforth, from the 3rd to the 14th years after planting). The following data were collected.

#### Fruit yield

The fruit was collected on the same day for all the treatments, when the fruits were at a ripening index of 3–4, from violet to black according to Barranco et al. (2005). A harvesting machine was used to collect the fruit, after which the fruit was weighed (in kg) for each experimental unit. These measurements allowed us to calculate the annual (kg fruit ha^−1^ year^−1^) and accumulated fruit yields (kg fruit ha^−1^) for every treatment.

#### Fruit characteristics and oil content

The fruit characteristics were evaluated by sampling each row, cultivar, and density in triplicate. The average fruit weight was measured in over 100 fresh fruits. The samples were then dried in a forced-air oven at 105°C for 42 h. and weighted again to determine the moisture content. The oil percentage was measured in these samples using a NMR analyser Minispec NMS100 (Bruker Optik GmbH, Ettlingen, Germany) according to the method described by del Rio and Romero (1999).

#### Oil yield

Based on the fruit yield and the oil content, we calculated the annual (kg oil ha^−1^ year^−1^) and the accumulated oil yield (kg oil ha^−1^).

#### Alternate bearing

To study the influence of the cultivar and the tree density on the alternate bearing behavior of the cultivars, we estimated their alternate bearing index (ABI) from the 3rd to the 14th year after planting. The ABI index was calculated using the following formula (Pearce and Doberšek-Urbanc, 1967):

$$\begin{array}{l} ABI=\frac{1}{n-1}\overset{n}{\underset{n=1}{\sum }}|\frac{{\text{a}}_{\text{n }+1}\;-\;{\text{a}}_{\text{n}}}{{\text{a}}_{\text{n}+1}\;+\;{\text{a}}_{\text{n}}}| \end{array}$$

Where *n* is the year and a_n_ is the yield at harvest.

To further characterize the alternate bearing behavior of the different treatments, we also calculated the bienniality index (B; Monselise and Goldschmidt, 1982), which indicates the percentage of occasions in which increasing or decreasing trends in yield were reversed between successive pairs of years. Both the ABI and B indexes range from 0 to 1, where 0 indicates no alternate and 1, maximum alternate bearing.

#### Vigor

The vigor of the adult trees was characterized by measuring their canopy depth and width (Connor and Gómez-del-Campo, 2013) during the 12th and 13rd winters after planting. The hedgerow volume and external surface area were calculated by considering the hedgerow as a rectangular parallelepiped; we used the following equations:

$$\begin{array}{l} \;Canopy\;volume\;(m^{3}\;ha^{-1})=(h-t)\times w\times r\times n\\ External\;surface\;area\;(m^{2}\;ha^{-1})=[2\;((h-t)\;\times r)\\ \;+\;(w\times r)]\times n \end{array}$$

Where *h* is the canopy height, *t* is the height above ground level maintained free of foliage to ease the management, *w* is the canopy width measured at breast height, *r* is the distance between trees, and *n* is the number of trees per hectare. Four measurements were taken per row. The vigor was further characterized by weighing the pruning wood of four trees per row during the winters of 2012 and 2014.

The ratio D/A of canopy depth (*h – t*) to free alley width (i.e., the row spacing minus the hedgerow width *w*) was calculated to evaluate the canopy illumination (Connor and Gómez-del-Campo, 2013).

#### Flowering

The flowering intensity was assessed by estimating the percentage of the canopy covered by inflorescences using a visual scale from 0 to 3, where 0 indicates no inflorescences; 1, = 33%; 2, 33–66%; and 3, >66%. This feature was evaluated at two heights (the upper and the lower half of the canopy) and in four trees per row during the 11th and 12th springs after planting. These estimates were later confirmed by evaluating the fruit distribution in the canopy.

### Statistical analysis

The effects of cultivar, tree density and year on the dependent variables—average annual fruit and oil yield (t year^−1^ ha^−1^), fruit weight (g), moisture (%), and oil content (%)—were examined using Friedman's test. This test was used because the data did not satisfy the requirements of parametric tests regarding normality, homogeneity of variance, or sphericity. The means were compared using Dunn's test with a Bonferroni adjustment at *P* = 0.05 (Demšar, 2006). Analysis of variance (ANOVA) was performed on the accumulated fruit and oil production based on a randomized complete block design; subsequently, the means were compared using Tukey's test at *P* = 0.05. Linear regression was used to evaluate the relationships between the accumulated production (of fruit and oil) and the number of years after planting. Likewise, the variables for yield and for vigor (canopy volume and surface area) and the hedgerow length were regressed against tree density. Finally, we calculated the coefficient of determination (*R*^2^) and the coefficient of determination adjusted for degrees of freedom (*R*^2^*a*).

One-way repeated measures were used to analyze the olive vigor, ABI, and B indexes because these data satisfied the sphericity requirement according to Mauchly's test. When appropriate, these data were transformed to be inverse or logarithmic. The flowering intensity and its distribution throughout the canopy were analyzed using a non-parametric Kruskal-Wallis test, after which Dunn's test (*P* = 0.05) was used to rank the cultivars based on their mean values. Statistical analyses were performed using Statistix software (Version 10; Statistix, Tallahassee, FL).

## Results

### Long-term evaluation of olive cultivars in SHD systems

Over 14 years, we monitored the relative performance of five olive cultivars that were widely used in SHD systems; in this study, the tree density was 1975 trees ha^−1^. During this period, the annual average fruit production of the cvs. “Arbequina,” Arbequina IRTA-i·18, “Arbosana” and “Koroneiki” ranged from 12.2 to 14.2 t ha^−1^; there was no significantly difference in production (*P* < 0.05) between these cultivars (Supplementary Figure 1A; Table 1). In contrast, “Fs-17” consistently showed the lowest production, with yields approximately half as large as for the other cultivars (6.2 t ha^−1^).

**Table 1:** **Annual (t ha^**−1**^), average (t ha^**−1**^ year^**−1**^), and accumulated (t ha^**−1**^) fruit yield for the five evaluated olive cultivars growing in SHD conditions in southern Spain**.

**Year after plantation/harvest season**														
**Cultivar**	**3**	**4**	**5**	**6**^a^	**7**	**8**	**9**	**10**	**11**	**12**	**13**^b^	**14**	**Average**^c^ **(t ha**^−1^ **year**^−1^**)**	**Accumulated**^d^ **(t ha**^−1^**)**
	**2002/03**	**03/04**	**04/05**	**05/06**	**06/07**	**07/08**	**08/09**	**09/10**	**10/11**	**11/12**	**12/13**	**13/14**		
Arbequina i-18	14.8	14.0	20.1	6.2	8.5	13.2	9.6	10.3	16.0	15.1	7.2	22.8	13.2a	157.9ab
“Arbosana”	17.2	9.0	19.4	7.4	17.4	9.0	15.3	10.8	20.4	15.0	10.6	18.7	14.2a	170.1a
“Arbequina”	16.6	17.2	21.6	6.3	8.0	9.9	10.5	10.9	15.3	13.3	8.9	19.4	13.1a	157.8ab
“Koroneiki”	20.7	6.1	12.5	4.5	14.8	10.1	13.6	8.6	20.1	9.9	9.1	16.5	12.2a	146.6b
“Fs-17”	4.4	2.8	8.8	0.7	10.9	4.6	7.8	4.0	14.4	5.1	7.2	3.8	6.2b	74.4c
Average^c^	14.7ab	9.8bc	16.5ab	5.0c	11.9abc	9.3abc	11.4abc	8.9abc	17.2a	11.7abc	8.6bc	16.2ab		

a *Severe frosts during the winter affected the tree growth*.

b *High temperatures (>30°C) during the bloom period affected the fruit set*.

c *Means with the same letter are not significantly different according to Friedman test followed by Dunn's comparison adjusted by Bonferroni at P = 0.05*.

d *Means with the same letter are not significantly different according to ANOVA followed by Tukey test at P = 0.05*.

Although there were no significant differences in average yield between the four most productive cultivars (“Arbequina,” Arbequina IRTA-i·18, “Arbosana,” and “Koroneiki”), we found significant differences in their accumulated fruit production (Supplementary Figure 1B; Table 1). The cv. “Arbosana” showed the largest accumulated fruit yield (170.1 t ha^−1^), followed by Arbequina IRTA-i·18, “Arbequina” and “Koroneiki.” There were significant differences (*P* < 0.05) between “Arbosana” and “Koroneiki” and between the cultivar “Fs-17” and all other cultivars (*P* < 0.001). This cultivar consistently had the lowest average as well as accumulated fruit production throughout the study period (Table 1).

Regarding the alternate bearing behavior of the cultivars, “Fs-17” also showed the most irregular production according to both the B and ABI indexes. “Arbequina” and Arbequina IRTA-i·18 had the most constant yields (Table 2). The information provided by the ABI and B indexes allowed us to rank the cultivars from greatest to least alternate bearing behavior as follows: “Fs-17” ≥ “Arbosana” = “Koroneiki” ≥ Arbequina IRTA-i·18 = “Arbequina” (Table 2).

**Table 2:** **Fruit characteristics, alternate bearing and vigor indexes for the five evaluated olive cultivars growing in SHD conditions in southern Spain during 14 years**.

**Cultivar**	**“Arbosana”**	**“Arbequina”**	**Arbequina i-18**	**“Koroneiki”**	**“Fs-17”**
Fruit weight (g)	1.7b	1.8b	1.8b	1.1c	2.8a
Fruit moisture (%)	58.0b	57.9b	58.4ab	55.0c	59.9a
Oil content^*^ (%)	17.8b	17.3b	17.6b	18.8a	18.8a
ABI^a^^c^	0.30b	0.20c	0.23c	0.33b	0.47a
B^b^	0.90ab	0.58d	0.67cd	0.85bc	0.95a
Tree height (m)	3.9	4.2	4.3	4.1	3.9
Tree width (m)	1.7	1.8	1.8	1.8	2.0
Canopy volume (m^3^ha^−1^) × 10^3^	14.1c	16.8a	16.8a	15.9b	16.3ab
External surface (m^2^ha^−1^) × 10^3^	21.0c	23.0a	23.4a	22.2b	21.7bc
Pruning wood (t ha^−1^)	10.5b	17.6a	15.5a	14.2a	17.4a
Canopy depth/free alley (D/A)	1.7	2.0	2.0	1.9	2.0

* *Fresh weight*.

a *Alternate bearing index (ABI) from the 3rd to the 14th year after planting, which was calculated according to Pearce and Doberšek-Urbanc (1967)*.

b *Bienniality index (B) from the 3rd to the 14th year after planting, which was calculated according to Monselise and Goldschmidt (1982)*.

c *Within each row, means with the same letter are not significantly different according to Friedman test followed by Dunn's comparison adjusted by Bonferroni at P = 0.05; or according to one-way repeated measures followed by Tukey's test at P = 0.05. No letter was added to any value when no significantly different pairwise comparisons were detected*.

“Fs-17” had the highest average fruit weight and moisture percentage (averaging 2.8 g and 59.9%, respectively), while “Koroneiki” showed the lowest values for these variables (averaging 1.1 g and 55.0%, respectively). The remaining cultivars formed a homogenous group between these two endpoints (Table 2). “Fs-17” and “Koroneiki” consistently had the highest oil contents (both averaging 18.8%), while “Arbequina,” Arbequina IRTA-i·18 and “Arbosana” had lower values (17.3–17.8%) with no significant differences between them (Friedman test; *P* > 0.05; Table 2).

There were no significant differences in average oil production between cultivars, with the exception of “Fs-17,” which had the lowest average production (Supplementary Figure 1C; Table 3). In terms of accumulated oil yield, “Arbosana” was the most productive cultivar, followed by “Koroneiki,” Arbequina IRTA-i·18, and “Arbequina” (Supplementary Figure 1D; Table 3). For this variable, there were significant differences between “Arbosana” and both Arbequina IRTA-i·18 and “Arbequina” (Friedman test; *P* < 0.05). The oil yield of “Koroneiki” was intermediate and showed no significant differences compared to “Arbosana,” Arbequina IRTA-i·18 or “Arbequina” (Table 3).

**Table 3:** **Annual (t ha^**−1**^), average (t ha^**−1**^ year^**−1**^), and accumulated (t ha^**−1**^) oil yield for the five evaluated olive cultivars growing in SHD in southern Spain**.

**Year after plantation/harvest season**														
**Cultivar**	**3**	**4**	**5**	**6**^a^	**7**	**8**	**9**	**10**	**11**	**12**	**13**^b^	**14**	**Average^c^(t ha^−1^ year^−1^)**	**Accumulated^d^(t ha^−1^)**
	**2002/03**	**03/04**	**04/05**	**05/06**	**06/07**	**07/08**	**08/09**	**09/10**	**10/11**	**11/12**	**12/13**	**13/14**		
Arbequina i-18	2.6	2.0	2.9	1.3	1.9	2.8	1.9	2.0	2.5	2.7	1.1	3.3	2.2a	26.8b
“Arbosana”	2.8	1.7	4.3	1.4	3.4	1.9	2.6	2.1	3.2	2.3	1.5	2.8	2.5a	29.9a
“Arbequina”	2.5	2.5	3.0	1.3	1.7	2.2	2.2	2.1	2.5	2.3	1.4	2.9	2.2a	26.3b
“Koroneiki”	3.8	1.1	2.6	0.9	3.1	2.2	2.8	1.7	3.3	1.9	1.5	2.6	2.3a	27.3ab
“Fs-17”	0.7	0.4	1.3	0.2	2.3	1.1	1.9	0.9	2.4	1.0	1.2	0.6	1.2b	13.9c
Average^c^	2.4abc	1.5bcd	2.8a	1.0d	2.4abc	2.0abcd	2.3abcd	1.8abcd	2.8ab	2.0abcd	1.3cd	2.4abc		

a *Severe frosts during the winter affected the tree growth*.

b *High temperatures (>30°C) during the bloom period affected the fruit set*.

c *Means with the same letter are not significantly different according to Friedman test followed by Dunn's comparison adjusted by Bonferroni at P = 0.05*.

d *Means with the same letter are not significantly different according to ANOVA following of Tukey test at P = 0.05*.

The accumulated fruit and oil production of the five cultivars increased linearly over time (*R*^2^ > 0.85; *P* < 0.001; Supplementary Figures 1B,D; Tables 1, 3). Thus, we did not observe any decrease in the annual average production of oil for any cultivar over the 14 years of evaluation. However, when we evaluated the distribution of the crop in the hedgerows, we determined that it was consistently concentrated in the upper half of the canopy for all five cultivars (Kruskal-Wallis test; *P* < 0.05; Table 4A). This feature was assessed by estimating the distribution of the flowers in the canopy and later confirmed based on the fruit distribution. The D/A values were ~2 for all the cultivars; “Arbosana” had the lowest value, with D/A = 1.7 (Table 2).

Table 4**Differences in flowering intensity between the upper and the lower half of the tree canopy for the five evaluated cultivars (A) and the cv. “Arbequina” at nine tree densities (B). Flowering intensity was assessed by estimating the percentage of the canopy covered by inflorescences using a visual scale from 0 to 3**.

**Table:** 

**(A) Flowering intensity**	**Cultivars**				
	**Arbequina i-18**	**“Arbequina”**	**“Arbosana”**	**“Fs-17”**	**“Koroneiki”**
Upper canopy	1.6a ± 0.11	1.4a ± 0.07	2.1a ± 0.08	1.2a ± 0.07	2.4a ± 0.10
Lower canopy	1.0b ± 0.08	0.8b ± 0.10	1.2b ± 0.05	0.8b ± 0.11	1.1b ± 0.08
*P***-**value	<0.001^*^	<0.001^*^	<0.001^*^	<0.001^*^	<0.001^*^

**Table:** 

**(B) Flowering intensity**	**Density (trees ha**^−1^**)**								
	**780**	**909**	**952**	**1143**	**1203**	**1481**	**1569**	**2000**	**2254**
Upper canopy	1.7a ± 0.21	1.5a ± 0.19	1.9a ± 0.17	1.7a ± 0.16	1.7a ± 0.17	1.5a ± 0.13	1.6a ± 0.21	1.4a ± 0.19	1.8a ± 0.28
Lower canopy	1.5a ± 0.26	0.8a ± 0.20	1.0b ± 0.23	1.1a ± 0.21	1.2b ± 0.14	1.0b ± 0.22	0.8b ± 0.22	0.8b ± 0.14	0.9b ± 0.17
*P***-**value	0.455	0.056	0.015^*^	0.057	0.036^*^	0.046^*^	0.0394^*^	0.0473^*^	0.023^*^

* *Significant differences according to a Kruskal-Wallis Test (P < 0.05)*.

We also compared the vigor of the olive cultivars. “Arbosana” was significantly (*P* < 0.05) more compact in shape, followed by “Fs-17” and “Koroneiki.” In contrast, “Arbequina” and Arbequina IRTA-i·18 had the highest canopy volumes and surface areas. To further characterize the vigor of the cultivars in SHD systems, we weighed the pruned wood in two years. The results showed that “Arbosana” produced 10.5 t ha^−1^ of pruning wood while the rest of the cultivars produced between 14.2 and 17.6 t ha^−1^, with no significant differences between the cultivars (*P* > 0.05). In other words, “Arbosana” produced ~35% less pruning wood than the other cultivars (Table 2).

### Effects of tree density on the long-term performance of the “Arbequina” cultivar

To assess the optimum hedgerow spacing in SHD olive orchards, we evaluated the performance of the cv. “Arbequina” to nine plantation densities ranging from 780 to 2254 trees ha^−1^ over 14 years. During this time, the fruit characteristics of “Arbequina” were stable and were not affected (*P* > 0.05) by the tree density. Regardless of the number of trees per ha, the average fruit weight was ~1.95 g, the moisture was around 57%, and the oil content relative to fresh weight was ~19% (Table 5).

**Table 5:** **Fruit characteristics and alternate bearing and vigor indexes for “Arbequina” at nine densities in southern Spain during14 years**.

**Density (trees ha^−1^)**	**780**	**909**	**952**	**1143**	**1203**	**1481**	**1569**	**2000**	**2254**
Fruit weight (g)	1.9	2.1	2.0	1.9	1.9	1.9	1.9	2.2	2
Fruit moisture (%)	57.4	56.6	57.1	57.0	57.5	57.1	57.1	57.2	56.9
Oil content^*^(%)	18.8	19.1	19.3	19.3	19.1	19.1	19.1	19.2	19.1
ABI^a^^c^	0.20ab	0.20ab	0.18bc	0.18bc	0.20ab	0.23a	0.19ab	0.17bc	0.14c
B^b^	0.70	0.70	0.70	0.70	0.70	0.80	0.67	0.72	0.72
Tree height (m)	3.8	3.9	4.0	4.0	3.9	4.0	4.0	4.1	4.2
Tree width (m)	1.9	1.9	2.0	1.9	1.9	1.8	1.8	1.8	1.9
Tree volume (m^3^ tree^−1^)	13.1a	11.9b	12.6ab	10.7c	10.1c	8.9d	9d	7.6e	7.9e
Hedgerow volume (m^3^ ha^−1^) × 10^3^	10.2f	10.8f	12e	12.2e	12.1e	13.2d	14.2c	15.2b	17.8a
External hedgerow surface (m2 ha^−1^) × 10^3^	14.1g	14.7g	15.9f	16.7e	16.9e	18.4d	19.8c	21.3b	24.3a
Pruning waste (t ha^−1^)	10.5	11.9	12.0	13.2	13.7	16.3	14.8	15.4	14.8
Row space (m)	5.7	5.5	5.2	5.0	4.7	4.5	4.2	4.0	3.5
Canopy depth/free alley (D/A)	0.9	1.0	1.1	1.2	1.2	1.4	1.6	1.8	2.3

* *Fresh weight*.

a *Alternate bearing index (ABI) from the 3rd to the 14th year after planting, which was calculated according to Pearce and Doberšek-Urbanc (1967)*.

b *Bienniality index (B) from the 3rd to the 14th year after planting, which was calculated according to Monselise and Goldschmidt (1982)*.

c *Within each row, values with the same letter are not significantly different according to Friedman test followed by Dunn's comparison adjusted by Bonferroni at P = 0.05; or according to one-way repeated measures followed by Tukey's test at P = 0.05. No letter was added to any value when no significantly different pairwise comparisons were detected*.

The average annual production increased linearly with the tree density, ranging from 7.1 to 12.9 t ha^−1^ for fruit and from 1.3 to 2.5 t ha^−1^ for oil yield (Supplementary Figures 2A,C; Table 6). Consequently, the accumulated production increased over time as a function of tree density, and the relationship significantly fit a linear regression (*R*^2^ > 0.85; *P* < 0.001; Table 6; Supplementary Figures 2B,D). Differences in tree density accounted for up to 86% of the accumulated oil production per ha during the study period; the accumulated oil yield ranged from 16.1 t ha^−1^ for the lowest density (780 trees ha^−1^) to 29.9 t ha^−1^ for the highest (2254 trees ha^−1^; Table 6). The flowers—and therefore the crop—were consistently concentrated in the upper half of the canopy for all plantation densities (Kruskal-Wallis test; *P* < 0.05; Table 4B), with the exception of the two lowest densities (708 and 909 trees ha^−1^) and 1143 trees ha^−1^. The two lowest densities were the only ones that showed D/A ≤ 1 (Table 5).

**Table 6:** **Annual (t ha^**−1**^), average (t ha^**−1**^ year^**−1**^), and accumulated (t ha^**−1**^) fruit and olive yield for “Arbequina” at nine densities 14 years after planting in southern Spain**.

**Year after plantation/harvest season**															
	**Density (Trees ha^−1^)**	**3**	**4**	**5**	**6**	**7**	**8**	**9**	**10**	**11**	**12**	**13**	**14**	**Average**^c^ **(t ha**^−1^ **year**^−1^**)**	**Accumulated**^d^ **(t ha**^−1^**)**
		**2002/2003**	**03/04**	**04/05**	**05/06**	**06/07**	**07/08**	**08/09**	**09/10**	**10/11**	**11/12**	**12/13**	**13/14**		
**Fruit (t ha**^−1^**)**	780	5.8	3.9	6.9	6.3	5.6	7.3	6.5	5.9	8.6	8.9	5.5	13.6	7.1d	84.8e
	909	6.8	3.6	7.9	6.9	6.1	7.3	7.9	6.8	7.7	8.9	5.5	14.9	7.5d	90.3e
	952	7.0	4.9	8.8	7.2	6.8	8.9	7.5	6.2	8.7	9.6	5.5	13.8	7.9cd	95.0de
	1143	8.1	4.9	9.6	8.0	7.3	7.9	7.7	7.3	9.7	10.6	5.9	15.3	8.5bcd	102.4cd
	1203	7.5	5.7	9.6	7.3	6.8	11.4	8.5	8.5	11.2	10.3	5.0	16.3	9.0bcd	108.0c
	1481	9.0	6.6	10.7	9.0	7.9	10.3	7.6	7.3	11.8	11.6	4.2	16.9	9.4abc	113.0c
	1569	8.8	8.0	11.9	8.9	7.7	12.1	11.0	8.5	13.6	12.8	7.0	16.9	10.6ab	127.2b
	2000	10.9	9.3	12.0	9.0	9.3	11.7	11.0	9.8	16.1	10.4	7.7	18.8	11.3ab	135.9b
	2254	10.4	8.3	14.4	11.1	10.9	12.1	12.2	10.5	17.9	15.3	10.6	21.4	12.9a	155.0a
	Average	8.3e	6.1bcde	10.2abc	8.2bcde	7.6de	9.9abcd	8.9abcde	7.9cde	11.7ab	10.9ab	6.3e	16.4a		
**Oil (t ha**^−1^**)**	780	0.9	0.8	1.2	1.3	1.3	1.6	1.4	1.2	1.4	1.7	0.9	2.3	1.3d	16.1f
	909	1.1	0.7	1.5	1.5	1.4	1.6	1.7	1.4	1.2	1.7	0.9	2.5	1.4cd	17.4ef
	952	1.2	1.0	1.8	1.5	1.5	2.0	1.6	1.2	1.4	1.8	0.9	2.3	1.5cd	18.4de
	1143	1.4	1.1	1.8	1.8	1.7	1.7	1.6	1.4	1.6	2.0	0.9	2.7	1.6bcd	19.8cd
	1203	1.2	1.2	2.1	1.5	1.4	2.4	1.8	1.7	1.8	1.9	0.8	2.7	1.7bcd	20.5cd
	1481	1.5	1.4	2.1	1.9	1.8	2.2	1.5	1.5	1.9	2.1	0.7	2.7	1.8bc	21.7c
	1569	1.4	1.8	2.1	1.9	1.8	2.6	2.3	1.7	2.2	2.4	1.1	2.7	2.0ab	24.1b
	2000	1.6	2.2	2.3	1.9	2.1	2.5	2.2	2.0	2.7	1.9	1.3	3.2	2.2ab	26.0b
	2254	1.6	1.9	2.5	2.4	2.6	2.7	2.5	2.1	3.1	2.9	1.8	3.6	2.5a	29.9a
	Average^c^	1.3ef	1.3def	1.9abcd	1.8bcdef	1.7bcdef	2.2ab	1.8abcde	1.6cdef	1.9abcde	2.1abc	1.0f	2.8a		

*^a^Severe frosts during the winter affected the tree growth*.

*^b^High temperatures (>30°C) during the bloom period affected the fruit set*.

c *Means with the same letter are not significantly different according to Friedman test followed by Dunn's comparison adjusted by Bonferroni at P = 0.05*.

d *Means with the same letter are not significantly different according to ANOVA followed by Tukey test at P = 0.05*.

Remarkably, we did not detect any progressive decrease in annual production over the study period for any of the density treatments (Table 6). The variability in yield between consecutive years was primarily due to the biennial cycle of the “Arbequina” cultivar. The most dramatic variations occurred in response to climatic stresses such as frost (in 2004–2005 and 2011–2012) and unusually elevated temperatures during bloom time (in 2012; Table 5). The alternate bearing behavior of “Arbequina” was not correlated with tree density (Table 5); neither the ABI nor the B indices had a significant relationship with tree density (Table 5). For instance, the highest density treatment, with 2254 trees ha^−1^, had the lowest ABI (0.14) and therefore weaker alternate bearing behavior. In contrast, the lowest density treatment, with 780 trees ha^−1^, showed an intermediate ABI value (0.20), and the intermediate treatment with 1481 trees ha^−1^ had the largest ABI (0.23) and therefore the strongest alternate bearing behavior (Table 5).

**Figure 1:**
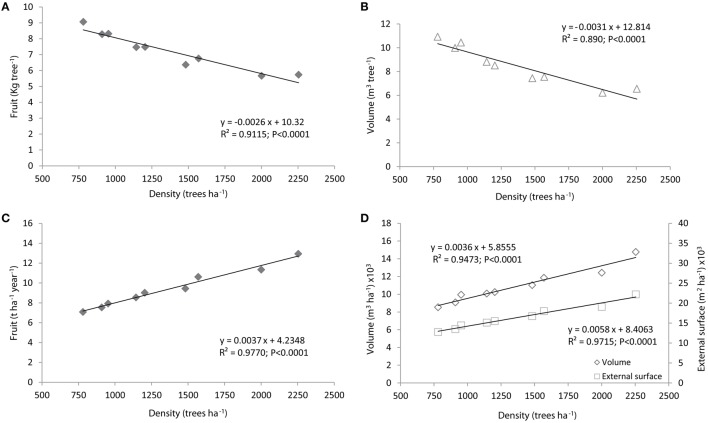
**The annual fruit production (A) and canopy volume (B) per tree are compared to the annual fruit production (C) and hedgerow surface and volume per hectare (D), both as functions of the olive tree density for the cultivar “Arbequina.”** Lines represent linear regression of the variables A, B, C, and D over tree density. The points represent the average values per olive tree or surface area of orchard over 14 years. Nine tree densities ranging from 780 to 2254 trees ha^−1^ were evaluated in this study.

The vigor (measured as canopy volume) and the yield per tree were negatively linearly correlated with the tree density (Figures 1A,B; Table 5). In contrast, the hedgerow volume, surface area, and production per hectare showed the opposite trend, increasing with tree density (Figures 1C,D; Table 5). Comparing the lowest and highest density treatments (780 and 2254 trees ha^−1^) showed that the volume of the trees decreased by ~40% while the volume and the external surface of the hedgerow increased by ~75 and 72%, respectively (Table 5). The pruning waste per hectare increased with tree density but only until intermediate tree densities (1481 trees ha^−1^), after which it remained stable, with a value close to 15 t ha^−1^ (Table 5).

## Discussion

### Long-term evaluation of olive cultivars in SHD systems

Since the inception of SHD olive orchards, there has been controversy regarding their management, productivity, lifespan, and profitability. In recent years, several studies have examined these features; however, they largely focused on young SHD olive orchards, given the recent popularity of these systems (Hermoso et al., 2004; Pastor et al., 2007; Camposeo and Godini, 2010; Larbi et al., 2011, 2012; Connor et al., 2012; Farinelli and Tombesi, 2015; Trentacoste et al., 2015a). Thus, no long-term characterizations of olive SHD orchards have been published. To address this gap in knowledge, we characterized the performance of various olive cultivars and the most frequent SHD plantation designs in field trials that lasted 14 years. The trials were located in the world's main olive-producing region, Andalusia, in southern Spain.

Over the study period, we evaluated the performance of three traditional olive cultivars commonly used in SHD systems, “Arbequina,” “Arbosana,” and “Koroneiki,” along with the popular clonal selection Arbequina IRTA-i·18 (Tous et al., 1998) and the new olive cultivar “Fs-17” (Fontanazza et al., 1998).

Seven years after planting, the preliminary results of these trials highlighted the early bearing of the cultivars in the study, with the exception of “Fs-17.” In addition, these results emphasized their high oil yields and showed subtle differences between the oil content and alternate bearing behavior of the various cultivars. The study also noted the stability and cultivar-specificity of the fatty acid profiles of the oils, which did not vary significantly between years (De la Rosa et al., 2007). Most of these early observations were later supported by other studies of young SHD orchards (Camposeo and Godini, 2010; Godini et al., 2011; Larbi et al., 2011; Tous et al., 2011; Farinelli and Tombesi, 2015; Trentacoste et al., 2015a).

Our 14 year study is the first to demonstrate the favorable long-term adaptation of “Arbequina,” Arbequina IRTA-i·18, “Arbosana” and “Koroneiki” in SHD systems. The 12 crops produced by these cultivars were highly productive, with an average oil yield of almost 2.3 t ha^−1^. More importantly, we note that their average production did not decrease over time. We also confirmed two early observations: first, the absence of significant difference between “Arbequina” and its clone selection Arbequina IRTA-i·18; and second, the different alternate bearing behaviors among the cultivars, with “Arbequina” and Arbequina IRTA-i·18 showing the most stable production, followed by “Arbosana,” “Koroneiki,” and “Fs-17.” Few studies have focused on alternate bearing behavior of olive (Rallo et al., 1993; Lavee, 2006; Larbi et al., 2011), suggesting that it should be systematically evaluated. This fact is particularly striking given that the olive biennial bearing is, along with climatic stresses, the cause of the greatest differences in production between consecutive years.

### Relative performance of the cultivars

The outstanding performance of “Arbosana” in SHD systems (Camposeo and Godini, 2010; Larbi et al., 2011; Tous et al., 2011; Rosati et al., 2013) is leading to its widespread use in orchards around the world, to the extent that it has begun to challenge “Arbequina” as the olive cultivar of choice for SHD systems. In our experiments, “Arbosana” and “Arbequina” had similar oil contents, but “Arbosana” had the largest accumulated oil yield (29.9 t ha^−1^) 14 years after planting. “Arbosana” also had the lowest vigor, as determined based on the canopy volume and external canopy area; it produced ~35% less pruning wood than “Arbequina.” These two cultivars, but especially “Arbosana,” coupled high productivity with a compact shape because of their higher branching efficiency, defined as the number of branches per unit of trunk cross-sectional area (Rosati et al., 2013). Branching frequency has been shown to be positively correlated with thinner bearing limbs, resulting in a larger number of potential fruiting sites per unit of trunk weight and thus higher yield efficiency (Rosati et al., 2013).

Despite their similarities, “Arbequina” and “Arbosana” differ greatly in their ripening time; “Arbequina” ripens in November, while “Arbosana” ripens in January–February (Barranco et al., 2000, 2005). This feature should be taken into account when planting “Arbosana” in areas with winter frost occurrence because frost damage is especially severe when fruits are green-yellow (Barranco, 2010). In addition, to avoid frost damage, “Arbosana” is often harvested too early, before reaching its maximum oil content, and this can lead to lower than expected oil yields.

The cv. “Koroneiki” was also favorably adapted to SHD conditions in terms of yield. It produced as much accumulated oil as “Arbequina” even though its fruit production was lower. “Koroneiki” has been described as highly productive, with high oil content and low to medium vigor (Barranco et al., 2000). Field observations in commercial orchards have confirmed the higher vigor of “Koroneiki” hedgerows compared to “Arbequina” and “Arbosana.” In addition, “Koroneiki” often presents vertical lignified top branches that are prone to damage during mechanical harvesting (Todolivo S.L, *personal communication*). Our characterization of the canopy volume and external surface did not reflect the higher vigor of “Koroneiki” observed in the field. In addition, the amount of pruning waste only distinguished the lower vigor of “Arbosana” compared to the rest of the cultivars. The assessment of cultivar vigor might not be trivial for fully-grown hedgerows because the tree height and width are determined by pruning (to fit the size of the straddle harvester, with a height of ~2.7–3.0 m and a width of 1.3 m) rather than by tree architecture. Thus, at this stage, it is likely that only large differences in vigor between cultivars (such as for “Arbosana”) can be correctly assessed. We note that the pruning waste, which affects the entire tree, could be a more reliable proxy for adult hedgerow vigor than the tree height and width.

Finally, the generally favorable adaptation of “Arbequina,” “Arbosana,” and “Koroneiki” contrasted with the poor performance of “Fs-17.” For instance, the average yield of “Fs-17” was half as large as that of the other cultivars, which agrees with Tous et al. (2008). High fruit production but substantial vigor and a spreading growth habit (which are not desirable features in SHD systems) were also previously reported for “Fs-17” (Camposeo and Godini, 2010). Although “Fs-17” had elevated oil content, these results suggest that “Fs-17” is not a suitable cultivar for SHD systems under the experimental conditions in this study.

We also note that in our study, “Fs-17” was highly susceptible to the fungus *Alternaria alternata* during a 2 year outbreak, while the remaining cultivars barely showed any symptoms of the disease (Moral et al., 2008). The microclimatic and agronomic conditions of SHD systems are highly conducive to airborne diseases. Thus, the differential resistance of the cultivars to major pests and diseases should also be taken into account when establishing an SHD orchard, especially in areas where *Colletotrichum* spp., the causal agent of olive anthracnose, is endemic (Moral et al., 2012). In addition, the numerous wounds in the branches caused by the mechanical harvester result in a greater risk of infections from wound-associated pathogens such as *Botryosphaeriaceae* species and the bacterium *Pseudomonas savastanoi* pv. *savastanoi* (Moral et al., 2010).

### Influence of tree density on the long-term performance of the “Arbequina” cultivar

We planted olive hedgerows of the cultivar “Arbequina” at nine densities ranging from 780 to 2254 trees ha^−1^; we achieved these densities by reducing the row spacing from 5.7 to 3.55 m and the tree spacing from 2.25 to 1.25 m. Preliminary results of this trial were reported 7 years after planting (León et al., 2007).

After 14 years of evaluation, two early observations were reinforced. First, the no influence of tree density on the characteristics of cv. “Arbequina,” including the biennial bearing behavior and the fruit oil content, moisture and average weight. Larbi et al. (2012) also described that tree density did not affect the biennial bearing behavior and the fruit oil content of this cultivar. However, they reported a negative correlation between tree density and fruit size that was not observed in our study. Similarly, Trentacoste et al. (2015a) reported a slight negative correlation, which was only significant in one out of four crops in their study, between tree density and oil content that was not supported by our results and those obtained by Larbi et al. (2012). The effect of tree density on fruit quality could be variable and difficult to uncouple from the illumination problems associated with high tree densities; in apple trees, for example, the planting density has a relatively low influence on fruit quality (firmness, soluble solids, titratable acidity, or weight) although poor illumination affected the color of the fruits (Wagenmakers and Callesen, 1995; Widmer and Krebs, 2001). In olive trees, fruits developed under conditions of high light interception show different fatty acid and polyphenol profiles than fruits developed in the shade (Gómez-del-Campo and García, 2012; Connor and Gómez-del-Campo, 2013).

The second observation confirmed by this study was the persistence of the positive correlation between density and oil yield preliminary reported (León et al., 2007). In this study, the accumulated oil production per hectare significantly increased by 86% in the highest density treatment (2254 trees ha^−1^) relative to the lowest density treatment (780 trees ha^−1^). Conversely, the accumulated oil production per tree decreased by 37%. The canopy volume showed a similar inverse relationship, increasing by 75% per hectare but decreasing by 40% per tree in the highest and lowest density treatments, respectively. While this dynamic has been described in young SHD olive orchards and for other fruit crops, its persistence over the long term has been questioned for olives (Wagenmakers and Callesen, 1995; Wheaton et al., 1995; Widmer and Krebs, 2001; Hampson et al., 2004; Larbi et al., 2012; Trentacoste et al., 2015a). For instance, yield efficiency in apple trees began to decrease by the eighth year at high densities (~3200 trees/ha; Hampson et al., 2004). Conversely, in citrus trees, there was no consistent relationship between yield and tree density for 9–13 year-old trees (Wheaton et al., 1995).

**Figure 2:**
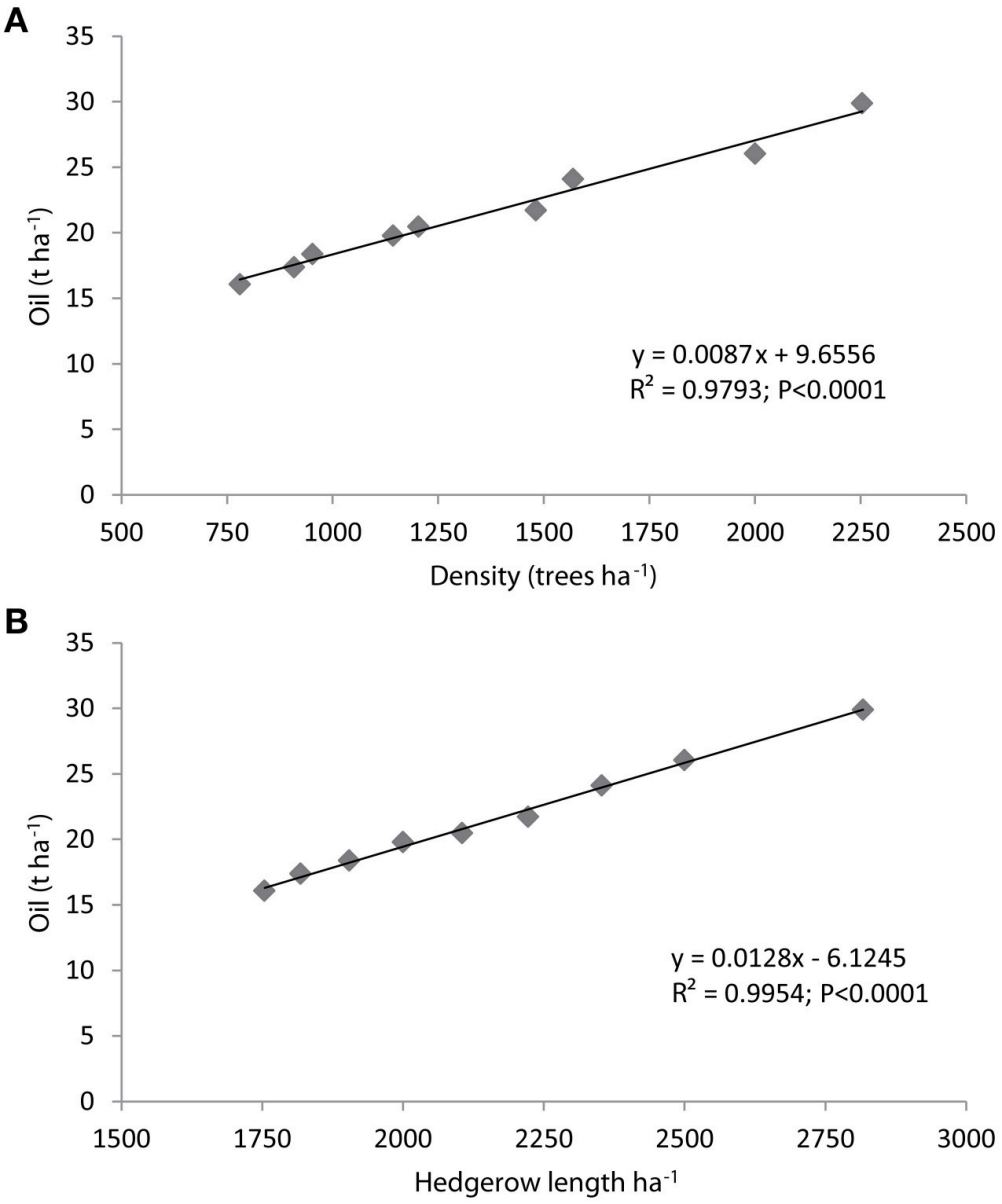
**Linear regression of accumulated oil per hectare over olive tree density (A) or the hedgerow length (B) over 14 years after planting the cultivar “Arbequina.”**.

We noted that the accumulated production per surface unit was highly correlated with the tree density but even better with the hedgerow length (*R*^2^ = 0.9793 vs. *R*^2^ = 0.9954; Figures 2A,B). In other words, the hedgerow length served as an accurate proxy of the accumulated yield of the adult hedgerows. This observation is especially remarkable given that the distance between trees does not affect the determination of the hedgerow length, which is basically determined by the row spacing. We hypothesize that if tree space is sufficiently reduced to rapidly lead to a solid hedgerow, the row spacing and therefore the length of the hedgerows will primarily determine the yield per ha. Trentacoste et al. (2015a) used the hedgerow length as a productive unit for SHD olive orchards; however, the tree spacing in their experiments was constant. In this study, the tree distance ranged from 1.25 to 2.25 m, but it had no influence on the accumulated yield 14 years after planting.

### Productive lifespan of olive hedgerows

One of the most important results of this study was the no decrease in the average annual yield of the SHD olive orchards 14 years after planting. Regardless of the cultivar and the tree density, the accumulated production of the hedgerows linearly increased as a function of time. Our results contradict those of previous SHD field trials in the same geographical area that showed declines in production seven or eight years after planting due to high vigor, shading, and limited ventilation (Pastor et al., 2007). The uncontrolled vigor of these hedgerows was probably due to their excessive irrigation dosage, which tripled the amount of water that was applied to our trials (6000 vs. 2000 m^3^ ha^−1^ year^−1^). Likewise, excessive fertilization could have also fostered the vigor of the trees; however, we cannot assess this hypothesis since the fertilization program for this previous trial was not fully detailed. The determination of soil characteristics, fertility levels, as well as the monitoring of “unintentional” sources of nutrients, such that coming from nitrate-polluted irrigation water, are critical determinants for a rational orchard management. Indeed, in an optimal climate and soil environment, managing the amount of water and fertilizers applied are crucial to avoid vigor problems that can compromise the lifespan of the hedgerow (Rallo et al., 2013; Connor et al., 2014).

Although the annual production of our hedgerows did not decrease over the time, we observed that by the end of the study period, the yield was consistently distributed in the upper part of the canopy (Table 4). This irregular pattern of fruit distribution in olive and other fruit crops is often related to illumination problems in the lower canopy (Wheaton et al., 1995; Farina et al., 2005; Connor et al., 2012). The optimum illumination for olive hedgerows occurs when the canopy depth equals the free alley width (D/A = 1; Connor and Gómez-del-Campo, 2013). Above this value, shading problems generally arise and trigger the accumulation of the production in the upper part of the canopy (Pastor et al., 2007; Trentacoste et al., 2015a). This feature has been reported even for very young olive hedgerows with D/A > 1 (Trentacoste et al., 2015a). In our study, only the two lowest density treatments of “Arbequina” (780 and 909 trees ha^−1^) and 1143 trees ha^−1^, with a row spacing ≥5 m and D/A ~1, showed a uniform distribution of the crop throughout the canopy. The only exception to this consistent pattern was “Arbequina” at 952 trees ha^−1^, which showed unequal distribution of the crop in the canopy with row spacing >5 m and D/A ~1. Even “Arbosana,” the cultivar with the lowest vigor, showed D/A > 1 (D/A = 1.7); hence, its production was concentrated in the upper part of the canopy.

The height of our hedgerows, which approached 4 m in all the treatments (cultivars and densities), could be the primary cause of this feature (Supplementary Figures 3A,B). The top branches were flexible enough not to be damaged by the straddle harvester, which worked at 2.6 m height (Supplementary Figures 3C,D). However, tall canopies mean that increasing proportions of the lower canopy receive little or no direct radiation, which is exacerbated by narrow alley width (Connor et al., 2012). The height of our hedgerows would require a minimum row spacing of 5.5 m to yield the optimum D/A = 1, a requirement that was only fulfilled by the two lowest density treatments.

While the D/A ratio seems to be a good proxy for hedgerow illumination, our results raise several questions. First, it is unclear how quickly accumulation of the production in the upper half of the canopy becomes significant when illumination problems arise; second, if this process is modulated by cultivar architecture and vigor; third, whether specific management practices such as pruning or deficit irrigation could avoid or minimize the effects of insufficient illumination within the alleys; and fourth, if specific pruning strategies could reverse these effects once they become pervasive in the hedgerows.

Finally, it is difficult to reconcile the apparent illumination problems of our hedgerows with the lack of decrease in oil production per hectare over time (even for the highest density treatments). It is not clear why we did not observe a correlation between these factors, even under favorable conditions (D/A > 1). However, we suggest that three main factors could account for our observations: first, the row spacing in our trials, which ranged from 3.55 to 5.70 m, was therefore not as restrictive as the 2.5 m evaluated by (Trentacoste et al., 2015a). Second, our study area had very good environmental conditions for the olive crop, which favored high yields; for example, the yields of “Arbosana” and “Koroneiki” in our study were almost twice as high as the average yields obtained in other locations (Camposeo and Godini, 2010; Larbi et al., 2011; Tous et al., 2011). Third, our pruning strategy was milder than in most other commercial hedgerows, which are heavily pruned to limit their height to 3 m to fit the straddle-harvester machine. Heavier pruning stimulates more vegetative growth and causes denser canopies and internal canopy shading, reducing fruiting capacity. Thus, it is possible that, due to our softer pruning, the upper canopy layers were able to compensate for the production of the layers with insufficient illumination. Tall hedgerows with narrow canopies have been proposed as promising orchards systems for apple tree in the near future (Robinson et al., 2013). In accord with this idea, there are pilot strategies in olive aimed to evaluate the combination of wider row spaces (5–5.5 m) and taller hedgerows (>4 m), given the progressive availability of harvesters able to straddle tall trees. These orchards represent lower initial costs because they have less hedgerow length per hectare, but also because of the same reason, lower production. However, it still needs to be evaluated whether taller hedgerows could be able to compensate this productive difference. Lastly, the remarkable inherent longevity and renovation capacity of olive trees compared to other fruit crops could contribute to this behavior. We note that Mediterranean agricultural landscapes are characterized by centuries-old, but still fully productive, olive orchards.

This study was the first to characterize the long-term performance of SHD olive hedgerows. However, additional comparative trials should be conducted in other geographical locations. This would allow us to establish general patterns and define optimal management strategies to ensure the long-term productivity of SHD olive orchards worldwide.

## Author contributions

Conceived and designed the experiments: DB and LR. Performed the evaluations: CD, DC, PM. Analyzed the data: JM, DC, CD, and DB. Wrote the paper: CD, JM, LR, and DB.

### Conflict of interest statement

The authors declare that the research was conducted in the absence of any commercial or financial relationships that could be construed as a potential conflict of interest. The reviewer TDJ declared a shared affiliation, though no other collaboration, with one of the authors JM to the handling Editor, who ensured that the process nevertheless met the standards of a fair and objective review.
