# Supplementary material for: Cultivar and Tree Density As Key Factors in the Long-Term Performance of Super High-Density Olive Orchards
Source: Front Plant Sci. 2016 Aug 23;7:1226. doi: 10.3389/fpls.2016.01226 (PMC4993835; doi:10.3389/fpls.2016.01226)
Supplement: Supplementary file 1 [file DataSheet1.pdf]

Supplementary Material

Cultivar and tree density as key factors in the long-term performance of super high-density olive orchards

Concepción M. Díez*, Juan Moral*, Diego Cabello, Pablo Morello, Luis Rallo, and Diego Barranco

- **Correspondence:** cmdiez@uco.es and jmoral@ucdavis.edu

1 Supplementary Figures

.

1.1 Supplementary Figures

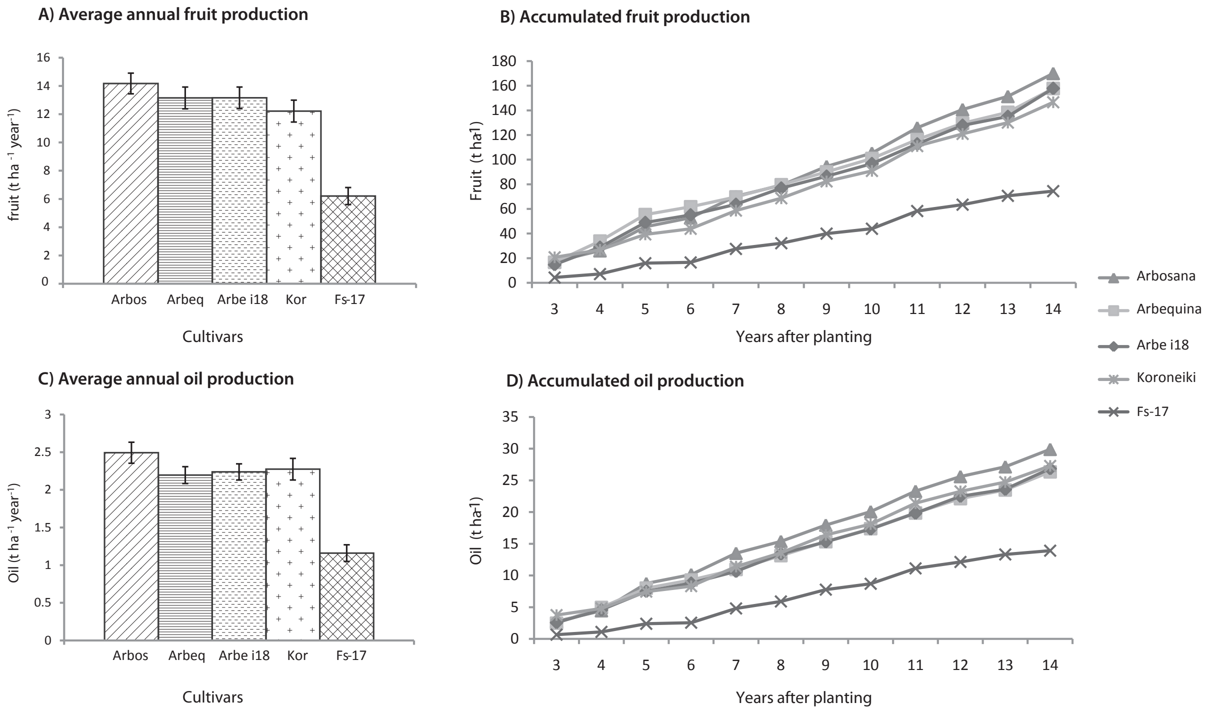

Supplementary Figure 1. Average annual and accumulated fruit and oil production for the five evaluated olive cultivars at 1975 trees ha^{-1} 14 years after planting.

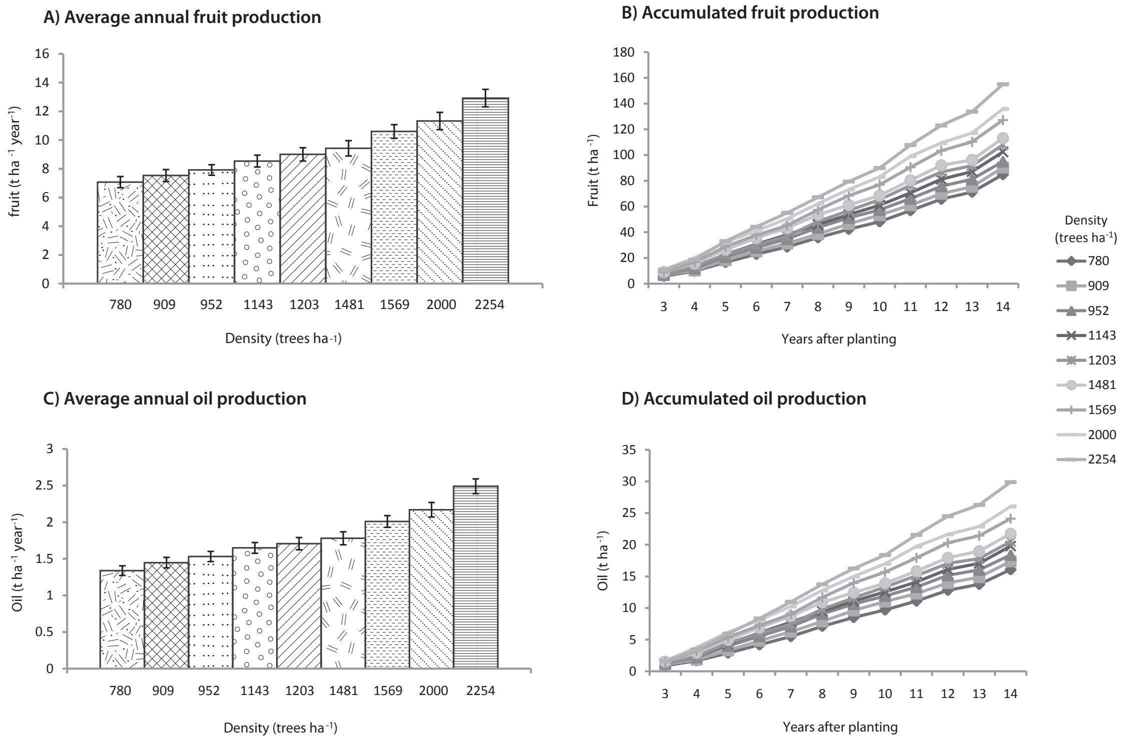

Supplementary Figure 2. Average annual and accumulated fruit and oil production per tree density for the cultivar 'Arbequina' 14 years after planting. Nine tree densities ranging from 780 to 2254 trees ha^{-1} were evaluated in this study.

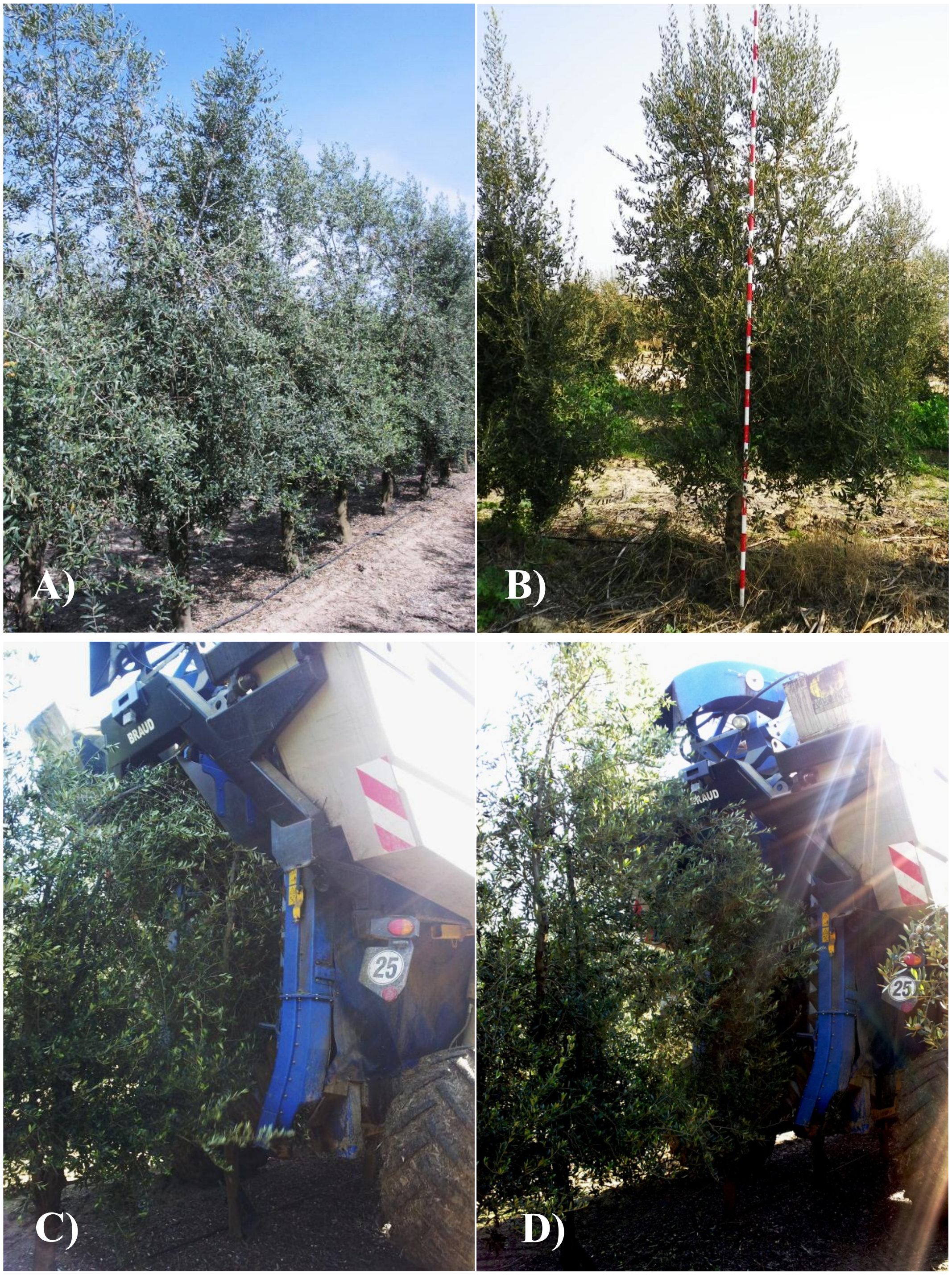

Supplementary Figure 3. A) General view of the ‘Arbequina’ hedgerow at 1975 trees ha^{-1}. **B)** ‘Arbequina’ tree at 909 trees/ ha^{-1}; the tree height for all the treatments was above 3.5 m (every band of the ranging pole measures 10 cm). However, the top branches were flexible **(C)** and therefore, they were not damaged by the straddle harvester, which worked at 2.6 m height **(D)**.
